# 20 years of research on giant viruses

**DOI:** 10.1038/s44298-025-00093-1

**Published:** 2025-02-11

**Authors:** Tressy Bosmon, Chantal Abergel, Jean-Michel Claverie

**Affiliations:** Aix–Marseille University, Centre National de la Recherche Scientifique, Information Génomique & Structurale, Unité Mixte de Recherche 7256 (Institut de Microbiologie de la Méditerranée, FR3479, IM2B, IOM), 13288 Marseille Cedex 9, France

**Keywords:** Viral evolution, Virus-host interactions, Virus structures

## Abstract

Some twenty years ago, the discovery of the first giant virus, Acanthamoeba polyphaga mimivirus (now *mimivirus bradfordmassiliense* species), paved the way for the discovery of more than 10 new families of protist-infecting DNA viruses with unexpected diversity in virion shape and size, gene content, genome topology and mode of replication. Following their brief description, we examine how the historical concepts of virology have held up in the light of this new knowledge. Although the initial emphasis was on the gigantism of the newly described viruses infecting amoebae, the subsequent discovery of viruses with intermediate virion and genome sizes gradually re-established a continuum between the smallest and largest viruses within the phylum *Nucleocytoviricota*.

## Introduction

Two people are traditionally credited for the discovery of the first virus, Dimitry Ivanovsky, who first demonstrated in 1892 the existence of an infectious agent not eliminated after filtration through a Chamberland filter-candle^1^, and Martin Beijerinck who proposed in 1898 that this agent (transmitting the tobacco mosaic disease) was of totally different nature than the microbes (bacteria) known at this time, and coined the term “virus”^2^. Unfortunately, his misinterpretation of viruses as “*contagium vivum fluidum*” (a non-particulate infectious fluid) only initiated the debates and confusion about the nature of viruses that were to mark the early years of virology^3^, until virus particles were finally revealed under the first electron microscope^4,5^. A formal concept of viruses was proposed by André Lwoff more than 65 years after their initial discovery.

His landmark articles^6,7^ proposed a small list of qualitative characteristics to distinguish viruses from non-viruses, as follows:

- Virions (i.e. the particles) possess only one type of nucleic acid, either DNA or RNA. Other agents (i.e. cellular organisms) possess both types.
- Virions are reproduced from their sole nucleic acid, whereas other agents are reproduced from the integrated sum of their constituents.
- Virions are unable to grow and undergo binary fission.
- Absence in the viruses of the genetic information for the synthesis of the Lipmann system, the system responsible for the production of ATP
- Viruses make use of the ribosomes of their host cells. This is defined as absolute parasitism.
- Viruses lack the information for ribosomal proteins, ribosomal RNAs and transfer RNAs syntheses.

Lwoff’s fundamental contribution was to insist that there could be no intermediary between viruses and non-viruses, an opinion far from unanimous in his day^7^. This is the meaning of his provocative aphorism “viruses are viruses”. This is also the underlying reason why none of the proposed criteria is quantitative, thus avoiding a threshold effect that would not have corresponded to the absolute demarcation he advocated between the viral and cellular worlds - a strict demarcation that has yet to be challenged, even if some of its initial criteria have been marginally called into question, as we shall see later on in this article.

Unfortunately, during the development of virology in the 20th century, as many new viruses were isolated in the context of human, animal, and plant diseases, researchers moved away from this rigorously defined paradigm and reintroduced more operational quantitative criteria adapted to their practice. This is how the property of ultrafiltrability, *i.e*. the ability of virions to pass through “sterilizing” filters (porosity <0.3 µm) became and has remained a major criterion, even though Lwoff had not retained it^7^ despite its historic nature^1^. Size fractionation using decreasing porosity filters remained the first sample processing step in most environmental studies, whether for virus isolation or metagenomic analyses^8–11^. The unjustified belief that viruses were necessarily the smallest microbes probably delayed the discovery of giant viruses by many years.

Following the characterization of many viruses (including phages), all exhibiting gene contents much smaller than cellular microbes, virologists also began to consider this feature – an expected consequence of their obligatory parasitism – as a basic trait of the whole viral world. We shall see that this too was a premature generalization.

Because of their small number of encoded proteins, most of which were structural components of the particles (and few genome replication enzymes for the most complex), the absence of any biosynthetic capacity also became considered a general feature of viruses, beyond the absence of energy metabolism already highlighted in Lwoff’s criteria^7^. In the wake of Lwoff criterion, any enzymatic function linked to protein synthesis was also considered prohibited. This again was a premature assumption.

The isolation and study of a large diversity of viruses in the 20^th^ century also led us to think that there was a clear partition between those replicating in the cytoplasm of their host, and those replicating in the nucleus. We will see that this frontier is now blurred for some of the newly characterized viruses.

Finally, the last century of virology has led us to think that viruses represented the ultimate bottom level of parasitism in the microbial world. This view has now changed with the discovery of virophages and several virus-associated mobile genetic elements^12,13^.

This article reviews the multiple new families of protist-infecting DNA viruses (Tables 1 and 2) most of which have been discovered over the last 20 years. Their unusual characteristics prompted a global reappraisal of the taxonomy of Nucleocytoplasmic Large DNA viruses^14^ (i.e. “NCLDV”) within the newly created phylum *Nucleocytoviricota*^15^, where they constitute new clades besides previously well-established families such as the *Poxviridae*^16^, the *Iridoviridae*^17^, the *Asfarviridae*^18^, and the *Phycodnaviridae*^19^. Amazingly, despite opening a whole new field in virology, none significantly violated Lwoff’s main discriminating criteria, published more than 65 years ago.

**Table 1:**
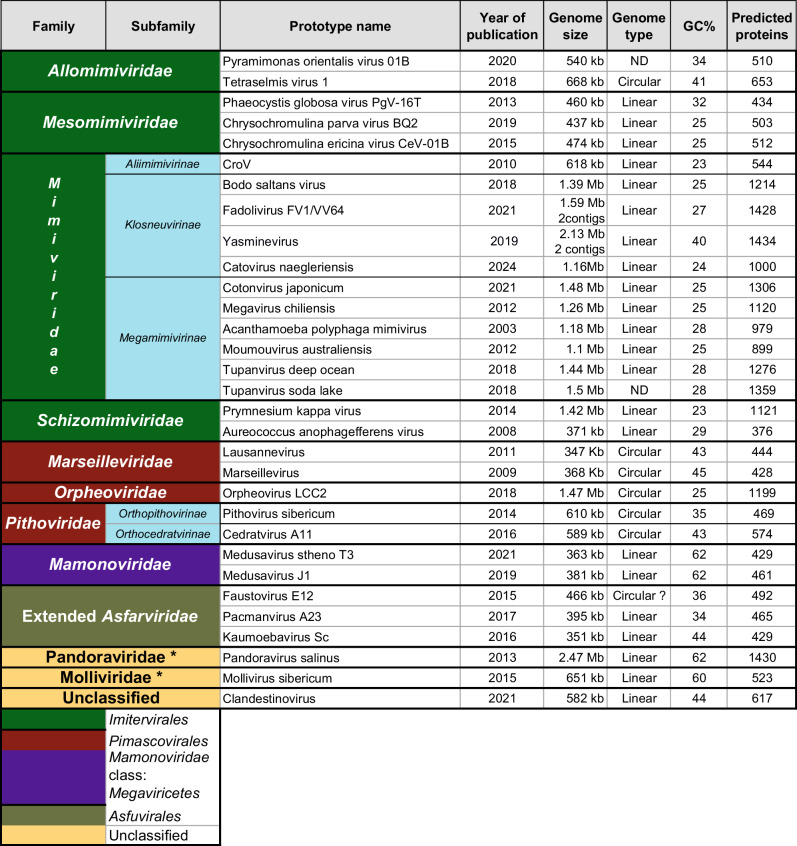
Large and Giant dsDNA Virus Prototypes Identified in the 21st Century

*Families not yet approved by ICTV.

**Table 2:**
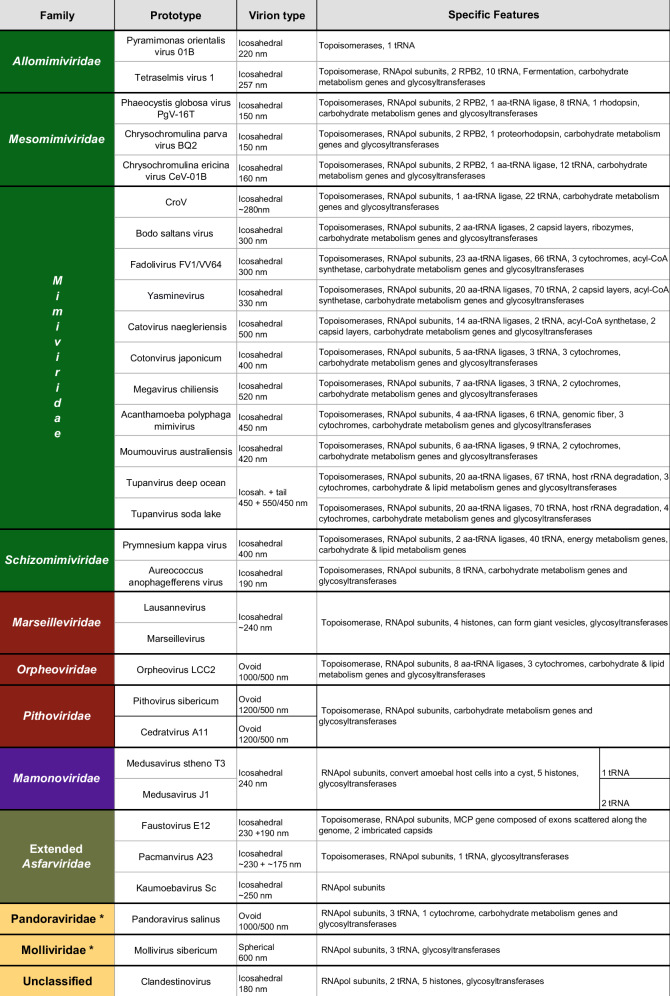
Characteristics of large and giant dsDNA viruses

^*^No ICTV classification.

## Mimivirus: the pioneer of the giant virus era

**Fig. 1:**
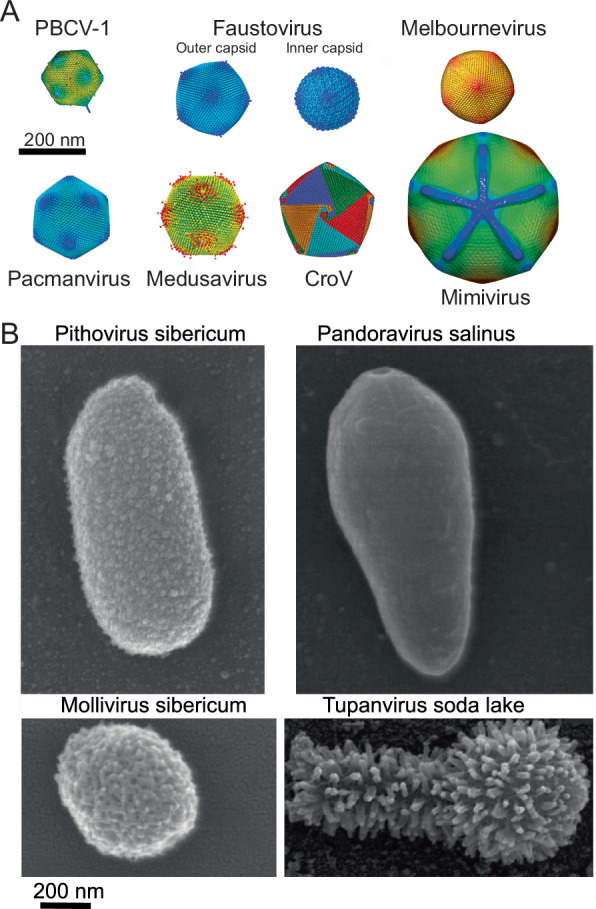
Images of virions. **A** Icosahedral virions structures determined by cryo-electron microscopy. PBCV-1 capsid (adapted from EMD-5384^125^ with permission), faustovirus outer (EMD-8144) and inner (EMD-8145) capsids^103^, melbournevirus capsid adapted from EMD-37169^126^ with permissions, pacmanvirus capsid (adapted with permission from ref. ^106^), medusavirus capsid (adapted form EMD-32073^127^ with permission), CroV capsid (adapted from EMD-8748^128^ with permission), mimivirus capsid (adapted with permission fromEMD-5039^129^). **B** Scanning electron microscopy images of some non-symmetrical virions. Pithovirus^85^, pandoravirus^68^ mollivirus^95^ and tupanvirus^28^ (courtesy from J. Abrahão).

For over 20 years, the record for the largest viral genome (331 kb) was held by Paramecium bursaria chlorella virus^20^ (i.e. PBCV-1), the prototype of a genus of viruses infecting *Chlorella*, a unicellular algae. This record was finally broken in 2004 by *Acanthamoeba polyphaga* mimivirus, an amoeba-infecting virus with particles large enough (0.75 µm in diameter, Fig. 1A) to be easily visible by light microscopy and a dsDNA genome of 1.181 Mb of unique sequence^21^. This first “giant virus” was determined to encode 979 proteins, 33 non-coding RNAs, and 6 tRNAs^22^, a genetic complexity comparable to some bacteria. It was also the first virus endowed with four aminoacyl-tRNA synthetases (basic components of the translation process) and five other translation factors, including a peptide chain release factor^23^, suggesting a possible origin by reductive evolution from an ancestral parasitic cell^24^. Yet, 70% of mimivirus-predicted proteins corresponded to ORFans, with no homologs in the cellular or viral world, making its origin puzzling and still heavily debated^25^.

Starting in 2009 with megavirus chilensis^26^, increasingly distant mimivirus relatives have been isolated across the world and in various environments establishing the ubiquity and high diversity of the initial *Mimiviridae* family that has now become the order *Imitervirales* composed of 3 additional distinct families^27^ (Tables 1 and 2). Mimivirus closest relatives are now grouped within the *Megamimivirinae* subfamily, the most spectacular members of which are the tupanviruses with genomes up to 1.516 Mb, encoding a complete set of aminoacyl-tRNA synthetases^28^ (Tables 1 and 2). The structure of the tupanvirus particles is also unique, exhibiting a large icosahedral capsid extended by a long tail making the total virion up to 2 µm in length^29^ (Fig. 1B).

All members of the subfamily *Megamimivirinae* present icosahedral capsids up to 450 nm that are highly complex, with an external layer of heavily glycosylated fibrils of variable lengths associated with a set of different proteins^30–32^ (Fig. 1A). Virally encoded glycosylation machineries synthesize their complex clade-specific glycans^33^ (Table 2). An internal lipidic membrane lines the capsid shell composed of the double jelly-roll fold major capsid protein (MCP), up to 5 MCP paralogs^34^, and other minor capsid proteins. An internal compartment, the nucleoid, contains the genome and the proteins needed to initiate the transcription of the early genes in the host cytoplasm. Upon infection, the particle internalized in the host cell vacuole delivers its nucleoid by opening a specific structure at one vertex called the “stargate”^35^, allowing the fusion between the virion and the vacuole membranes. This leads to the development of an organelle-like structure, the viral factory (VF) which has been recently proposed to be made by phase separation^36^. In the nucleoid, the mimivirus genome is organized into a long flexible, helical structure (30-nm diameter), made of proteins (unexpectedly homologous to GMC oxidoreductases) encasing the genome with the transcription machinery at the center of the genomic fiber^37^.

All the members of the *Mimiviridae* family (Table 1) appear to have linear dsDNA genomes flanked by long terminal repeat (TIR), up to 26 kb long for the *Megamimivirinae* subfamily^38^ (Claverie & al., in preparation). Within the phylum *Nucleocytoviricota*, this genome topology is shared with 3 other distant viral clades: the poxviruses (order *Chitovirales*), the asfiviruses (order *Asfuvirales*), and the chloroviruses (order *Algavirales*). As all these TIR-ending genomes have been shown to arbor covalently cross-linked (hairpin) ends^39–41^ it is tempting to suggest that this also applies to the *Mimiviridae* family. Within the whole *Imitervirales* order, we noticed the intriguing exception of the *Allomimiviridae* family, the only one known to include members with circular dsDNA genomes (Tetraselmis virus 1, and Dishui lake large algae virus 1, Table 1)^42,43^.

For most members of the *Imitervirales*, the host cell nucleus appears undisturbed by infection, except for members of the subfamily *Klosneuvirinae*, which induce its degradation in their hosts, the amoeba *Vermamoeba vermiformis*^34^ and the marine kinetoplastid *Bodo saltans*^44^. As of today, for all members of the subfamily *Klosneuvirinae* it has been proposed that an additional capsid shell was imbricated into the larger icosahedral capsid covered by fibrils.

Another surprising feature of members of the *Mimiviridae* family and the *Mesomimiviridae* is that their VF could be the target of parasitic infection by smaller viruses, called “virophages”^45–48^. Virophages have ~20 kb circular dsDNA genomes and virion size ~50 nm and are now classified in their own family, the *Lavidaviridae*^49,50^. They replicate in the giant virus VF, use the same regulatory elements as the giant virus (late promoter and hairpin rule), and depend on its replication machinery^51^. They can either interfere with the giant virus replication or be commensal^13,52,53^. The virophage mavirus infecting members of the genus *Rheavirus*, can integrate its genome into the cellular host’s genome (members of the genus *Cafeteria*). Upon infection by the associated giant virus, the virophage is reactivated, thus protecting the cellular host population^54^. Virophages can thus act as an antiviral defense for some protists. Virophages lacking their own major capsid protein gene can still propagate as episomes in the virion or integrate into the genome of their giant companion virus^47,48^.

The sequencing of several members of the *Megamimivirinae* subfamily led to the discovery of a new mobile genetic element, dubbed transpoviron. They consist in 7-kb-long dsDNA TIR-ending linear molecules encoding 6–8 proteins. Transpovirons mostly propagate as episomes within both the giant virus or virophage particles^12,53^, but are also found integrated in virophage or giant virus genomes.

## Marseillevirus: at the limit of viral gigantism

Following the discovery of mimivirus, searching for other acanthamoeba-infecting viruses led to the unexpected discovery of the unrelated marseillevirus in 2009^55^. It became the prototype of a rapidly expanding family, the *Marseilleviridae*, in the order *Pimascovirales* within the phylum *Nucleocytoviricota*. Members were isolated all around the world, in all types of environments by co-cultivation or identified through metagenomic analyses^56–59^. They have circular genomes up to ~380 kb and virions have icosahedral capsids ~250 nm in diameter (Fig. 1A). They all encode the main DNA-dependent RNA polymerase subunits (Table 2) and were first thought to be exclusively cytoplasmic viruses until the mass spectrometry-based proteomic analysis of the virion revealed that these enzymes were not loaded in the virions. During the early stage of their infectious cycle, marseilleviruses induce changes in the host nucleus to recruit the nuclear transcription machinery to their nascent VF and express early genes, including those coding for the viral RNA polymerase subunits. The nucleus integrity is then restored. The *Marseilleviridae* could therefore be an evolutionary intermediate between exclusively cytoplasmic giant viruses and nuclear viruses^60^. Another distinctive feature of members of the *Marseilleviridae* is the presence of histone genes corresponding to histone doublets H2B-H2A, miniH2B-H2A, and H3-H4, linked by a ∼20-amino-acid connector^56^. In vitro, the fused histones H2B-H2A and H3-H4 assemble into unstable nucleosomes resembling eukaryotic nucleosomes, but wrapping ~120 bp DNA. These histones are expressed late, are essential, and highly abundant in the viral particles, suggesting a chromatin-like genome organization in the capsid^61,62^. Since then, core histones have been identified in another family of viruses in the phylum *Nucleocytoviricota*, the *Mamonoviridae* (prototype: medusavirus*)*, which in addition to the 4 histones also encode an acidic protein resembling the linker histone H1^63^. The latter is expressed early, while the 4 core histones are expressed late and form in vitro tri-nucleosome arrays^64^. In contrast with members of the *Marseilleviridae*, medusaviruses transfer their genomes to the host nucleus to initiate DNA replication, while particle assembly and DNA packaging take place in the cytoplasm near the nucleus^63^. More recently clandestinovirus was isolated on *Vermamoeba vermiformis*. It presents some distant relationship with the *Mamonoviridae* and was also shown to encode the 5 histones, with a fused H2B/H2A, H3 and H4, and the linker histone H1. Clandestinovirus possesses a much larger genome (~580 kb) than the members of the family *Mamonoviridae* (~380 kb) and 65% of its genes are ORFans (Table 1). Clandestinovirus infectious cycle takes place in the host nucleus and neosynthesized virions are transferred in large vacuoles and then exported by exocytosis^65^.

## Pandoravirus

As the ubiquity, abundance, and unanticipated diversity of the mimivirus relatives were gradually revealed^27,47,66,67^, it wasn’t until 2013 that the prototypes of the new family *Pandoviridae* (not yet classified by ICTV) were discovered and characterized^68^.

Unexpectedly, two isolates were concomitantly amplified from samples collected from distant sites, immediately suggesting their ubiquity. Pandoravirus dulcis, isolated from a freshwater pond near Melbourne, Australia, and pandoravirus salinus, isolated from shallow sediments collected at the mouth of the Tunquen River on the Pacific Ocean coast near the Las Cruces marine station^68^ in Chile. After amplification on *A. castellanii* cell cultures, both isolates were found to multiply and form lawns of heterogeneous ovoid particles 1 µm long on average and 0.5 µm in diameter (Fig. 1B). Their replication cycles last 10–15 h, starting with the phagocytosis of individual particles and ending with the release of approximately a hundred new particles following host cell lysis.

Thin-section transmission electron microscopy imaging revealed oblong particles enveloped in a 70-nm-thick electron-dense shell, interrupted by an ostiole-like pore at one end (Fig. 1B). Delimited by an inner lipid membrane, the electron-lucent interior of the particle is devoid of visible substructure, in contrast to the compact nucleoids observed in most icosahedral viruses. This last feature is all the more astonishing given that the pandoravirus salinus genome remains the largest DNA sequence ever obtained for a virus, at 2.47 Mb plus large terminal repeats at both extremities^68^ (Table 1).

Despite such a genome size, largely overlapping that of many bacteria, referring back to Lwoff’s criteria, namely the absence of binary division, the absence of ribosomes, and the absence of genes encoding a translation apparatus, enabled pandoraviruses to be unambiguously classified as new members of the viral world^68^.

Since the discovery of the first two prototypes, more than a dozen pandoravirus strains have been isolated and characterized using *A. castellanii* as cellular host, from very diverse environments^69–74^. However, except in rare occurrences^75^ pandoraviruses have remained scarce in metagenomic data^76,77^ in contrast to *Mimiviridae*, perhaps due to biases in DNA extraction methods.

Isolated (and fully sequenced) pandoraviruses all have G + C rich (>61%) TIR-ending dsDNA linear genomes with sizes ranging from 2.47 Mbp (p. salinus) to 1.6 Mbp (p. massiliensis) (Table 1). Unexpectedly, a rigorous reannotation of the genomes combining transcriptomic, proteomic, and bioinformatic analyses revealed many non-coding transcripts and significantly reduced the set of protein-coding genes predicted by standard ab initio methods such a Genemark (e.g. from 2556 to 1430 for p. salinus, much less than the usual one gene/kb ratio)^70^. Such remarkably low coding density among viruses is due to unusually long UTRs.

All known pandoraviruses propagate through virions exhibiting very similar morphologies and sizes. In contrast with other giant virus families, their common (*i.e*. core) protein-coding gene content is a small fraction of their total gene content (e.g. 455 vs 1430 for p. salinus), while their pangenome appears as yet unlimited. If the proportion of multiple-copy genes (up to 55%) is higher in pandoraviruses than in other giant virus families, gene duplication cannot explain their remarkable genome size. The same is true for the estimated proportion of genes gained through horizontal transfer (approximately 15%). Together with the high proportion of ORFans in the pangenome, these findings led to the proposal that the (G + C) rich genomes of pandoraviruses are privileged sites of de novo gene creation^70^.

Despite their unprecedented genome size, the pandoravirus core gene content conspicuously lacks genes encoding essential functions such as DNA ligase, topoisomerases, and DNA sliding clamps suggesting that their replication requires host enzymes normally segregated in the nucleus. Surprisingly, even if the pandoraviruses encode a rather complete transcription apparatus (Table 2, RNA polymerase, mRNA-capping enzyme, several transcription factors), none of these are detected in the virion proteome. This again implies that the replication process cannot be initiated without involving the host transcription apparatus.

With the presence of several spliceosomal introns, the above features suggest that at least part of the pandoravirus genome is transcribed within or using functions recruited from the host nucleus. This is consistent with nucleus disruption observed 4 h post-infection in several cells^68,71^.

Another unique feature of pandoraviruses is the complete absence of any gene coding for a homolog of the major capsid protein (MCP), one of the most archetypal core gene among large DNA viruses (*Nucleocytoviricota*), often used as a reference for phylogenetic or environmental studies^78–80^. This probably made pandoraviruses difficult to identify in large-scale metagenomic studies. Interestingly, the absence of a MCP is not strictly linked to the non-icosahedral symmetry of the pandoravirus virions, as remote (structural) MCP homologs are encoded in the genome of poxviruses^81,82^ and mollivirus^83^.

Finally, one group has reported that one layer of the thick integument enclosing pandoravirus massiliensis might consist of cellulose^84^, while the viral genome does not appear to encode the corresponding synthesis pathway. This would be the first example of a virus “outsourcing” part of its capsid synthesis to its cellular host, a unique mechanism in the virosphere.

## Pithovirus

The first member of this genus, pithovirus sibericum, was isolated from a 30,000 years old sample of permafrost^85^. The oblong virions can reach 2 µm in length for 0.5 µm in diameter (Fig. 1B). One tip exhibits a cork organized as a honeycomb array. The virions are enclosed in an electron-dense integument made of parallel strips covered by oligosaccharides. As with all viruses that enter the cell by phagocytosis, the capsid’s inner envelope is covered by a lipid membrane. The transcription machinery encoded by the virus is loaded into the virions, enabling the infectious cycle to begin in the host cytoplasm within the viral factory. Three hours post-infection (pi), the virions are built from the apex cork, first as rectangular membrane-enclosed immature virions later transformed into mature oblong particles following the progressive addition of the external layer. Virions are then released by exocytosis. We noticed that pithoviruses uniquely lack a homolog of the DNA virion packaging ATPase, a key enzyme well conserved in other large dsDNA viruses. On the other hand, the 3’ end signal of their transcripts is a hairpin structure similar to that used by members of the *Mimiviridae* and *Iridoviridae* families^85^.

Many pithovirus relatives have been isolated around the planet^86,87^ and are frequently detected in metagenomic studies of various environments^88,89^. Among these relatives, a divergent branch emerged with cedratvirus presenting the same virions morphologies, but with a cork at each extremity of the virions^90,91^. All pithovirus relatives have been classified within the order *Pimascovirales*, constituting a separate clade besides the previously defined *Iridoviridae*, *Ascoviridae* and *Marseilleviridae* families^87^. This clade was initially proposed to include 3 families: *Pithoviridae*, *Orpheoviridae*, and *Cedraviridae*^92^. The latest taxonomical ICTV proposal is now separating all genera of pithovirus relatives into the suborder *Ocovirineae* comprising three families *Pithoviridae* (split into two subfamilies *Orthopithovirinae* and *Orthocedravirinae*), *Orpheoviridae*, and *Hydriviridae* (Claverie et al., ICTV release in preparation). Members of the family *Pithoviridae* all present rather small circular genomes in the range of 460–686 kb, thus decorrelated from the huge volume of the virions. *Verbamoeba vermiformis* infecting orpheovirus^93,94^ and hydrivirus^88^, assembled from metagenomic permafrost data, have much larger genomes up to 1.6 Mb genomes (Table 1). *Orthopithovirinae* subfamily members have larger genomes than *Orthocedravirinae* members due to the invasion of 2 main inverted repeats that have massively colonized their genomes^86^. As expected from viruses exclusively replicating in the cytoplasm, pithovirus particles carry the virus-encoded transcription machinery, albeit surprisingly, without a recognizable polyA polymerase homolog (Table 2).

## Mollivirus

The same permafrost sample from which the first pithovirus was isolated, also provided the prototype of the (proposed) *Molliviridae* family: mollivirus sibericum. The first member of this new family was thus revived from particles trapped in north-eastern Siberian permafrost for 30,000-years^95^. Following this initial discovery, no other mollivirus relative was reported until 5 years later, making them the most elusive members of the phylum *Nucleocytoviricota*. As the first modern isolate, called mollivirus kamchatka^96^, was isolated from a region with a subarctic climate, it was initially thought that molliviruses were confined to cryosol environments. This was recently disproved by the isolation of a new strain from marine sediments collected in Uranouchi Bay in Japan, a region with a rather warm climate (33.4197° N, 133.3580° E)^97^. Thus, like other members of the *Nucleocytoviricota* infecting *Acanthamoeba*, molliviruses, although rare, are probably ubiquitous.

Using electron microscopy, all known molliviruses have very similar particle morphologies and size: spherical particles (0.6–0.75 µm in diameter) surrounded by a 20-nm-thick electron-dense integument consisting of 3 layers and covered with a mesh of fibers (Fig. 1B). They all exhibit G + C-rich (>60%) linear dsDNA genomes in the [620kb-650kb] range, ending with ∼10-kb-long inverted repeats, predicted to encode about 500 proteins^95^ (Table 1).

As for other *Acanthamoeba*-infecting giant virus, mollivirus particles enter the host cytoplasm through phagocytosis, followed by a fusion of the virion internal lipid membrane with that of the phagocytic vacuole allowing the release of the particle content into the host cytoplasm. Using Edu labeling, mollivirus viral DNA entry into the cell cytoplasm and its migration to the nucleus was visualized using fluorescence microscopy^95^. Neosynthesized virions appeared in the extracellular medium 6 h pi without exhibiting the cell lysis described for other giant viruses^95,96^. Virion assembly is triggered on an open cisterna with a flat-pole and vesicles are recruited and open to form open curling ends on immature crescent-like particles with additional capsid layers. DNA-associated filaments accumulate in the VF and genome packaging takes place prior closure of the virions^95,98^. Along with pandoravirus, mollivirus is another paradoxical example of a virus encoding a transcription apparatus (Table 2), that is not loaded in its virion, making the initiation of its replication dependent on the host nucleus^95^. Accordingly, about 4% of the protein-encoding genes exhibited spliceosomal introns delimited by the canonical 5′-GT–3′-AG rule.

A comparative genomic study indicated a high proportion (64%) of predicted proteins without significant sequence similarity in the NCBI protein database. Among non-ORFan proteins (186), 83 had their best matches with pandoravirus proteins, 50 with an Acanthamoeba protein, 22 with proteins of other eukaryotes, and 18 with prokaryotic proteins^99^. Such inhomogeneity might result from horizontal gene transfers of unparalleled proportion in the phylum *Nucleocytoviricota*.

Despite the spherical shape of their virions and their phylogenetic affinity with pandoraviruses, molliviruses possess a well-recognizable double jelly-roll major capsid protein homolog (mollivirus sibericum: ml_347, accession # ALD62149). This protein, present in the virion, was found to be essential and might act as a scaffolding protein for integument biosynthesis^83^. Unexpectedly, the closest known homolog of this protein is also encoded in *Acanthamoeba* species (63% identical in *A. castellanii*), suggesting a recent gene exchange between mollivirus and its host, among many other documented examples of insertions events^100^.

Because of their significant morphological and gene content differences with pandoraviruses, and their large phylogenetic distance with other *Nucleocytoviricota* clades, molliviruses are proposed to be classified in their own *Molliviridae* family.

## Distant relatives of African swine fever virus

Using *Vermamoeba vermiformis* as an alternative host to isolate giant viruses led to the isolation of 8 members of a new lineage, the faustoviruses, distantly related to African swine fever virus^101^. Faustovirus E12 exhibits icosahedral virions ~200 nm in diameter (Fig. 1A) containing a 466 kb circular dsDNA genome (Table 1). Virions are internalized through phagocytic vacuoles, and their content is transferred to the host cytoplasm after membrane fusion of the virion’s inner membrane with the phagosome membrane. A donut-shaped viral factory surrounded by mitochondria appears 4–6 h pi. Neo-synthesized virions first accumulate at its periphery as empty capsids then loaded with DNA, gradually filling the cytoplasm with regularly aligned mature virions. Cell lysis occurs 18–20 h pi^101,102^. Like the African swine fever virus, faustovirus encodes 2 large polyproteins (220kDA and 60 kDa). The virion proteome comprises 162 proteins including homologs of capsid proteins, the conserved A32-packaging ATPase, RNA polymerase, transcription factors, and transcript maturation enzymes^101^, compatible with an entirely cytoplasmic infectious cycle (Table 2). A specific feature of faustoviruses is the presence of an additional, smaller ~175 nm icosahedral capsid imbricated into the ~260 nm icosahedral virions (Fig. 1A). The two capsid structures have been determined by cryo-EM^103^. In addition, the major capsid protein (MCP) is encoded by a 17 kb spanning gene containing 6 group I self-splicing introns conserved in all other isolates^102^. Kaumoebavirus-Sc was the second member of the extended *Asfarviridae* isolated on *Vermamoeba vermiformis*^104^. The virions exhibit icosahedral capsids ~250 nm in diameter enclosing a ~350 kb dsDNA genome first thought to be circular (Table 1). Further analyses of the genome of the KV-LCC10 strain revealed it was linear, flanked by TIR, with incompletely base-paired hairpin termini, suggesting this was also the case for all kaumoebaviruses, faustoviruses, and pacmanviruses^105^. The ~3 kb MCP-encoding genes exhibit between 2–5 type I introns. The infectious cycle is similar to that of faustoviruses.

Pacmanvirus A23 was isolated on *Acanthamoeba castellanii*^106^. The icosahedral capsids are 200 nm in diameter (Fig. 1A) and contain linear dsDNA genomes up to 420 kb (Table 1). The external capsids can be disrupted producing characteristic-shaped virions reminiscent of the Pac-Man video game character. The virions incorporate the components required for transcription (Table 2) and the cytoplasmic infectious cycle follows the same stages as faustovirus, but more rapidly. The capsid is transferred into the cytoplasm and changes morphology next to mitochondria, with the electron-dense inner compartment becoming rectangular. The VF is built next to the nucleus, and neosynthesized virions accumulate at its periphery in dense networks until they fill the cytoplasm. Cell lysis occurs at 8 hpi. Additional pacmanviruses have been isolated from diverse environments^107,108^ and faustovirus-like sequences are prevalent in hematophagous biting midges and their hosts^109^. Faustoviruses, pacmanviruses and kaumoebaviruses constitute distinct clades within the family *Asfarviridae*, distantly related to the original Asfarviruses^18^.

## Conclusion

Following the creation of more than 10 new families (Tables 1 and 2) required to classify the large number of protist-infecting viruses discovered in the last 20 years, one might have expected that the criteria for defining viruses established by Lwoff 60 years ago would have to be radically revised. Surprisingly, even though most of them feature novel characteristics, such as virion size and/or gene content that largely overlap those of the cellular domain, only a small number of the original criteria (recalled above) have been called into question. They are briefly discussed below.

### Reproduction from the sole genetic material

For Lwoff, a virus (more precisely, a virion) cannot be considered as an organism, but as a simple “box” whose only role is to carry the viral genome, the only element necessary for the cell to replicate the virus. All the viruses listed above clearly refute this view, as their infectious cycles could not proceed without involving complex macromolecular structures (e.g. inner membrane, “stargate”, “cork”, chromatin-like assemblages, etc…), as well as a variety of virally encoded enzymatic functions (e.g. transcription apparatus, nucleotide synthesis, complex carbohydrates) as well as several hundreds of other proteins of unknown functions. The ongoing studies of viral proteomes are gradually revealing the existence of viral metabolisms of unexpected complexity and diversity.

### Absence of “viral metabolism” and ATP synthesis

Virally encoded carbohydrate biosynthesis was first described for *Chlorella variabilis* NC64A cells producing hyaluronic acid once infected by PBCV-1^110^. Since then, it has been discovered that several viruses of the phylum *Nucleocytoviricota* encode complete glycosylation pathways enabling the biosynthesis of highly complex glycans independently from the host carbohydrate-processing enzymes^111^.

Lwoff’s criteria about the absence of viral ATP synthesis was more directly challenged by the discovery of virally-encoded key fermentation enzymes^42^, several enzymes of the TCA cycle^29,112^, and photosynthesis enzymes in some cyanophages^113,114^. Metagenomic analyses confirmed the widespread presence of such enzymes in viral populations^115,116^. In addition, lipid and sugar metabolisms, nutrient and transport enzymes, rhodopsins and cytoskeletal proteins, have been reported in several viral genomes and metagenomes (reviewed in ref. ^117^). Such genes can be classified into different categories. Those for which there are homologs in the host thus complementing its metabolism^42,112,117^, hijacked cellular genes that can be reprogrammed to assume additional, antagonist or new functions^37,118–121^, and viral genes absent from the host that provide new capabilities to the infected cell^115,122,123^.

### Viruses don’t do translation

Among Lwoff’s discriminating criteria, the translation process occupies a central place. Viruses are obligate intracellular parasites because they lack the ribosomes to synthesize their proteins. Despite the imposing size of their particles, even the largest virions (such as pandoravirus or pithovirus) do not carry ribosomes. They are therefore entirely dependent on the ribosomes they find in their host’s cytoplasm.

Nevertheless, some viruses isolated in recent years have undermined the criterion that they should not contain genetic information related to the translation apparatus. The most blatant counter-examples are to be found in the *Mimiviridae* family, most of which possess numerous tRNA genes, translation factors, and even more surprisingly, functional amino-acyl tRNA ligases^124^ (such as tupanvirus, which possesses a complete set^28^). The fact remains, however, that none of the recently discovered viruses possesses the genetic information required to synthesize functional ribosomes. Therefore, the absence of translation remains the prerogative of the viral world.

**Fig. 2:**
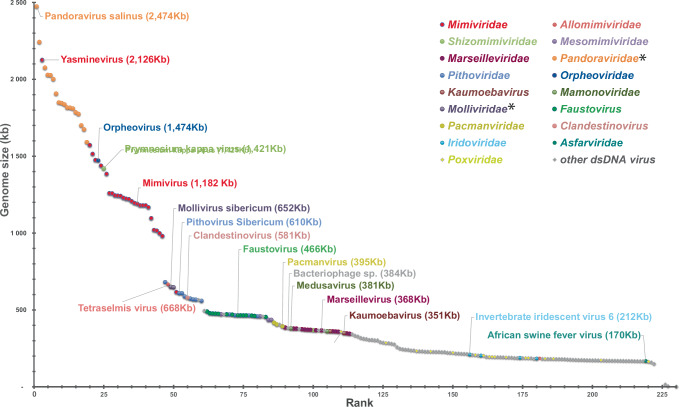
Distribution of viral genome sizes, illustrating the emergence of a continuum between giant and large dsDNA viruses. *Proposed family names not yet validated by ICTV.

Since the discovery of mimivirus, many members of new families of protist-infecting viruses have been isolated all over the planet. Most propagate through giant virions visible by light microscopy and exhibit genomes larger than 400 kb. Moreover, metagenomic studies have shown them to be highly diverse and widespread in all environments. The increase in the number of viruses with genomes larger than that of PBCV-1, the previous record holder, has also filled the gap between the chloroviruses and the almost 3 Mb genome pandoraviruses, restoring a continuum between the smallest and the largest dsDNA viruses (Fig. 2).

By shattering the paradigm of filterability that has governed virus detection for over a century, the discovery of mimiviruses has opened up a new chapter in virology that is likely to hold many more surprises, some of which might further challenge Lwoff’s historical criteria.

## Data Availability

No datasets were generated or analyzed during the current study.
